# The Scourge

**Published:** 1876-10

**Authors:** 


THE awful scourge which has invaded the seaports of Georgia the present year, in the form of yellow fever, calls for the most profound and thorough investigation as to the cause, and means of lessening its ravages in the future. Much of theory has been written, and also well-established facts have been published by medical observers. At the same time there are circumstances found to surround infected cities whose connection with the production of yellow fever exist alone in the imagination of the investigator.

Every suggestion made by medical men who have witnessed the prevalence of the disease, should not be taken for granted, without being sustained by indisputable facts. Some of them are not only wanting in this particular, but palpably contradictory of each other. For example, eminent physicians differ as to the importation of the disease and its contagiousness: and while one observer declares that it cannot exist without contiguity to the seacoast, and a proper direction of the wind to impregnate the air with saltish vapor, another declares that Memphis, at least three hundred miles by an air line from the sea, has been fearfully scourged by yellow fever. Again, opposite opinions are entertained as to the connection of malaria with its production, and also of the conditions or circumstances necessary to the production of this morbific agent.

Now, in view of all this, would it not be well to institute, at Savannah and Brunswick, investigations of the topographical surroundings, the nature and time of overflows by fresh water, etc.?

